# Dose effect of Actisaf Sc 47 yeast probiotic (*Saccharomyces cerevisiae*) supplementation on production, reproduction, and negative energy balance in early lactation dairy cows

**DOI:** 10.1093/tas/txad132

**Published:** 2023-12-14

**Authors:** Dana Kumprechtová, Héloïse Legendre, Romana Kadek, Valentin Nenov, Maxime Briche, Nizar Salah, Josef Illek

**Affiliations:** Nutrition and Feeding of Farm Animals, Institute of Animal Science, Praha Uhříněves, 104 00, Czech Republic; Phileo by Lesaffre, Marquette-lez-Lille, 59520, France; Clinic of Ruminant and Swine Diseases, University of Veterinary Sciences Brno, 612 42, Czech Republic; Phileo by Lesaffre, Marquette-lez-Lille, 59520, France; Phileo by Lesaffre, Marquette-lez-Lille, 59520, France; Phileo by Lesaffre, Marquette-lez-Lille, 59520, France; Clinic of Ruminant and Swine Diseases, University of Veterinary Sciences Brno, 612 42, Czech Republic

**Keywords:** dairy cow, energy use, milk yield, reproduction, *Saccharomyces cerevisiae*, yeast probiotic

## Abstract

The study evaluated the dose effect of dietary supplementation with yeast probiotic *Saccharomyces cerevisiae* (CNCM I-4407, 10^10^ CFU/g, Actisaf Sc 47; Phileo by Lesaffre, France) on production, energy metabolism, and reproduction in lactating dairy cows. About 117 multiparous Holstein cows from 3 to 60 d in milk held in a barn with an automatic milking system were enrolled in a randomized complete block design and blocked according to calving day, parity, and previous milk yield. The cows were assigned to a basal diet (15% CP, 22% starch) plus either 5 g (Y5 group, *n* = 39), 10 g (Y10 group, *n* = 39), or 0 g (CON, *n* = 39) of yeast probiotic, presented on top of concentrate fed in the robot. Milk yield and body weight were recorded daily, milk composition, and somatic cell count (**SSC**) every 2 wk, and body condition score (**BCS**) was estimated at days −14, 14, and 40 post-calving. Data were analyzed using a linear mixed model. The Y10 group showed an increased average daily yield of energy-corrected milk (**ECM**) over CON (+3.5 kg, *P* < 0.05) and Y5 (+0.8 kg). There were no significant differences between the groups in milk fat, milk protein, milk SCC linear score, milk urea, blood beta-hydroxy-butyric acid levels, and BCS. Body weight loss from 3 to 90 d in milk was numerically lower (13.8 kg) in Y5 than in CON (25.3 kg), and the success rate from the first insemination was the highest in YP5 and YP10 groups (39%) than in Control (26%). The yeast probiotic supplementation to early lactation high-producing dairy cows showed a clear effect of the high dose (10 g) on ECM milk production, although the lower dose (5 g) showed only numerical ECM production increase, both doses displayed better use of energy from the diet than the control and suggest a better resource efficiency.

## Teaser

The research demonstrates that dietary supplementation with yeast probiotic *Saccharomyces cerevisiae* has a significant impact on dairy cow performance, resulting in increased milk yield and improved reproductive performance. Furthermore, the study highlights the crucial role of rumen microbiota and the potential of precision feeding using probiotics to optimize production and reproduction in high-producing dairy cows.

## Introduction

Production and quality of ruminants are determined by the amount and nature of products derived from ruminal fermentation. Feeding a ruminant is above all feeding its rumen microbiota, which lives in symbiosis with the host animal (Julien et al., 2018). Profile and functioning of rumen microbiota depend on many factors such as diet (composition, structure), stage of production cycle, health status, and management. Ruminal microbiota can also be modified by probiotics.

Dietary supplementation with yeast probiotics has an effect on animal performance; in beef cattle, this is reflected in growth improvement (Hancock et al., 1994), while in dairy cows it is primarily increased milk yield (Stella et al., 2007; Desnoyers et al., 2009; Moallem et al., 2009; Doležal et al., 2012; Zaworski et al., 2014; Salvati et al., 2015; Kumprechtová et al., 2019).

Several modes of action of yeast probiotic *Saccharomyces cerevisiae* in the rumen have been suggested and investigated (Nisbet and Martin, 1991; Newbold et al., 1996; Callaway and Martin, 1997). Some researchers reported the ability of yeast to mitigate the ruminal pH decrease in cows fed high-carbohydrate diets (Bach et al. 2007; Marden et al., 2008; Desnoyers et al., 2009).

Improvements in feed efficiency and milk yield have been reported in response to yeast probiotic supplementation to dairy cows (Eastridge, 2006; Bruno et al., 2009; Desnoyers et al., 2009; Moallem et al., 2009; Bitencourt et al., 2011; Marsola et al., 2010; Doležal et al., 2012). In this context, Kumprechtová et al. (2019) found significantly lower serum NEFA and beta-hydroxy-butyric acid (**BHBA**) in the cows in peak lactation receiving yeast probiotics.

Efficient utilization of energy and protein from feed is a determining factor in improving the chances of successful reproduction in high-producing dairy cows. Julien et al. (2018) observed a positive effect of a yeast probiotic supplementation on the reproductive performance of dairy cows.

Dose-response studies of feeding yeast to lactating dairy cows are limited. Ferraretto et al. (2012) studied the addition of 0, 2, and 4 g/cow/d of live yeast to high-starch diets for dairy cows and observed higher milk fat content and total tract fiber digestibility with 4 g/cow/d than with the lower doses.

The objective of this study was to investigate the dose effect of dietary supplementation of yeast probiotic *S. cerevisiae* strain CNCM I-4407 in high-producing dairy cows in early and peak lactation on production and reproduction in association with negative energy balance. The goal of this study is in line with the approach to precision feeding using milking robots where not only the concentrate but also the probiotics can be dosed to cows´ individual performance, life stage, and feed intake.

## Materials and Methods

### Animals and Diets

Animal handling followed the European Union Directive 86/609/EEC (European Council Directive, 1986) on the protection of animals used for scientific purposes, the European Convention for the protection of vertebrate animals used for experimental and other scientific purposes (European Treaty Series, 1986), Act number 246/1992 Coll. of Laws of the Czech Republic on the protection of animals against cruelty as amended.

**Figure 1.**
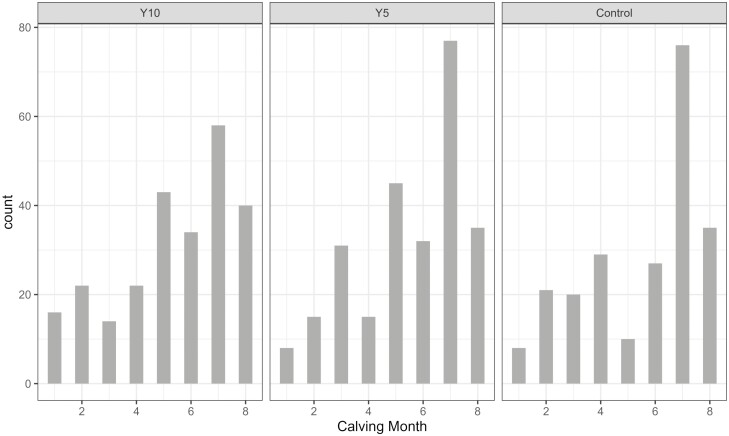
Calving distribution histogram. Y5 = Control Diet + 5 g/d of yeast probiotic (5 × 10^10^ CFU/cow/d); Y10 = Control Diet + 10 g/d of yeast probiotic (10 × 10^10^ CFU/cow/d); Count = a number of calvings per month.

The study was performed in a dairy herd with 500 Holstein cows in the Czech Republic. In the trial, 117 multiparous cows were included. The barn was equipped with an automated milking system (Lely A3, NL). There were eight robots, each per 55 cows. The cows were allocated in three dietary treatments: Yeast Probiotic 5 g (Y5), Yeast Probiotic 10 g (Y10), and Control (CON), based on parity, previous lactation milk yield, and calving date (Table 1), housed in 6 separate pens. The treatments Y5, Y10, and CON were represented in each of the 6 pens. There were 39 cows in each treatment. Body condition score (**BCS**) at calving was between 3.25 and 3.5 in all the cows included in the study and was not used as a covariate. The cows were fed a partial mixed ration (**PMR**) in the feed bunk and pelleted concentrate in the milking robot (Table 2). The calving distribution is provided in Figure 1. The concentrate allowance was based on milk yield (if milk < 21 kg = 2 kg concentrate, milk < 41 kg = 2.6 kg concentrate, milk < 50 kg = 4 kg concentrate, milk < 55 kg = 4.5 kg concentrate, milk > 55 kg = 5 kg concentrate). Every cow received 200 g propylene glycol per day from 0 to 20 d in milk (**DIM**).

**Table 1:** Randomization of the experimental cows (mean values and standard errors of the mean)

Item	Y5^1^ (*n* = 39)	Y10^2^ (*n* = 39)	Control (*n* = 39)	SEM	*P* value
Average parity rank	2.46	2.79	2.90	0.152	NS^3^
Average milk production/previous lactation (305 d), kg	9,800	10,411	10,253	262.5	NS

^1^Y5 = Control Diet + 5 g/d of yeast probiotic (5 × 10^10^ CFU/cow/d).

^2^Y10 = Control Diet + 10 g/d of yeast probiotic (10 × 10^10^ CFU/cow/d).

^3^NS: statistically non‐significant.

**Table 2:** Composition of the diet

Ingredients	September	October	December	January
Maize silage, kg	23.0	25.0	23.5	20,0
Clover-grass silage, kg	—	20.0	9.0	11.0
Whole crop rye silage, kg	—	—	10.4	10.0
Barley straw, kg	0.5	0.5	0.3	0.2
WDGS (maize), kg	—	—	4.2	1.5
Pea silage, kg	18.4	—	—	—
Brewer´s grains, kg	3.7	—	—	—
Rapeseed meal, kg	2.0	2.8	1.8	1.9
Soybean meal, kg	0.7	0.7	-	-
Ground wheat + barley 1:1, kg	—	—	2.3	2.5
Ground wheat + triticale 1:1, kg	—	1.6	—	—
Ground wheat, kg	1.5	—	—	—
Wheat flakes, kg	2.0	—	—	—
Corn flakes, kg	—	1.3	2.0	2.2
Fat C:16 90%, kg	0.4	0.4	0.4	0.45
Mineral concentrate^1^, kg (MK Sebranice with biotin, Se)	0.5	0.5	0.5	0.5
Concentrate given in robots^2^, kg (average amount/cow)	4.9	4.9	4.9	4.9
Total fresh matter (FM)/cow, kg	57.60	57.70	59.30	55.15
Total dry matter (DM)/cow, kg	25.00	25.48	25.66	25.59
Dry matter, %	39.61	40.24	40.80	39.92
Crude protein (on DM basis), %	15.93	13.37	15.39	14.73
ADF (on DM basis), %	21.94	22.44	19.78	21.77
NDF (on DM basis), %	32.95	34.02	30.86	32.49
Starch (on DM basis), %	22.14	20.58	23.80	22.62
Ash, %	7.07	6.71	6.35	7.04

^1^Mineral concentrate composition: VPC (39.2%), salt (18.8%), NaHCO_3_ (25%). Calcium (157 g/kg), phosphorus (9.2 g/kg), sodium (140.0 g/kg), magnesium (34.0 g/kg, sulfur (11.6 g/kg), chlorine (110.0 g/kg), copper (690.0 mg/kg), zinc (3,220.0 mg/kg), manganese (2,070 mg/kg), cobalt (12.0 mg/kg), iodine (46.0 mg/kg), selenium (40.0 mg/kg, of which selenomethionine 30.0 mg/kg), vitamin A (414,000.0 IU/kg), vitamin D3 (55,000.0 IU/kg), vitamin E (as alphatocoferol) (2,024.0 mg/kg), and biotin (50.0 mg/kg).

^2^Composition of pelleted concentrate given in robots: corn (34.9.0%), rapeseed meal (27.6%), soybean meal (15.4%), wheat (15.4%), sugar (5.1%), limestone (1.1%), and rapeseed oil (0.5%).

The Y5 and Y10 cows received 50 and 100 g, respectively, of a premix of yeast probiotic (strain CNCM I-4407, 10^10^ CFU/g; Actisaf Sc 47; Phileo by Lesaffre, Marcq-en-Baroeul, France) and wheat flour as a carrier (10% yeast probiotic + 90% wheat flour), using an automatic feed dispenser in the robot, on the top of pelleted concentrate, at one dose per day, from 3 to 60 DIM. This premix was pelleted with no heat treatment.

### Measurements and Sample Collection

#### Milk yield

Milk yield was recorded every day (DIM11 to DIM100) for every cow included in the trial, using the herd management system Afifarm (S.A.E. Afikim, Israel). Energy-corrected milk (**ECM**) was calculated using the equation of Sjaunja et al. (1990):


$$\begin{array}{l} ECM(kg/d)=\frac{Milk(kg/d)\times [0.383\times \;fat(\%)+0.242\times protein(\%)+0.7832]}{3.1138} \end{array}$$

Fat-corrected milk (**FCM**) was calculated using the equation of Gaines (1928):


$$\begin{array}{l} 4\%FCM(kg/d)=Milk(kg/d)\times [0.4+15\times fat(\%)] \end{array}$$

#### Milk composition

Milk samples were collected individually and every 2 wk for 24 h consecutive hours (all the samples per one cow per 24 h were mixed and analyzed as a pooled sample), and analyzed for fat, protein, and lactose percentages, SCC, and urea. The milk samples were preserved with bronopol (2-bromo-2-nitro-1,3-propanediol) and analyzed at the official milk testing laboratory (LRM Brno, CZ). The concentrations of fat, crude protein, and urea were analyzed by infrared spectrophotometry and somatic cell count by flow cytometry using Combi Foss (Foss Electric, Hillerød, Denmark). Somatic cell scores were calculated using the following equation (Ali and Shook, 1980):


$$\begin{array}{l} SCS=log_{2}\left(\frac{SCC}{100}\right)+3\;\;\;\;\; \end{array}$$

#### BCS and body weight

BCS was estimated for each cow included in the study approximately at days −14 pre-calving, and 14 and 40 post-calving. BCS was evaluated using a scale from 1 (emaciated) to 5 (overweight) in increments of 0.25 according to Edmonson et al. (1989). Body weight (**BW**) was measured daily in the milking robots.

#### Blood ketone bodies

BHBA levels in blood were measured by an on-farm test, using the device WellionVet BELUA (Efekta, s.r.o., CZ), on days 5 and 21 post-calving.

#### Estimation of feed intake

Total daily dry matter intake (**DMI**) was estimated every 2 wk until 105 DIM, using the following equation based on Parity (coefficient equal to 1 for multiparous, 0 for primiparous), milk Energy (MilkE, Mcal/d), BW (kg), BCS and DIM from NASEM 2021 (National Academies of Sciences, Engineering, and Medecine; de Souza et al., 2019):


$$\begin{array}{l} DMI(kg/d)=[(3.7+Parity\times 5.7)+0.305\times Milk\;E+0.022\times BW+(-0.689+Parity\times (-1.87))\times BCS]\times [1-(0.212+Parity\times (-0.136))\times e^{\left(-0.053\times DIM\right)}] \end{array}$$

Milk E was calculated as follows using NRC 2001 (National Research Council) equation:


$$\begin{array}{l} Milk\;E(Mcal/d)=Milk(kg/d)\;\times [0.0929\times fat(\%)+0.0547\times protein(\%)+0.192] \end{array}$$

Daily DMI of PMR only was also estimated, every 2 wk until 105 DIM, using the model provided by NASEM (2021) based mainly on measured milk performances (milk yield, fat, and protein contents). Firstly, the energy requirements for the maintenance and lactation of each cow were calculated based on the following equations:

$0.080/kg\;\;\;BW^{0.75}$ for maintenance,

(0.0929×FC%+0.0563×PC%+0.192)×MY for lactation.

Energy requirements for gestation were assumed to be 0.04 or 0.1 Mcal/d, respectively, for DIM < 50, 50 < DIM < 100, 100 < DIM < 150). For growth, the energy required was considered at 0.53 during the second lactation or 0 Mcal/d for higher lactation. Secondly, energy provided by the concentrated feed was estimated based on the amount of concentrate distributed to cows based on milk yield (from 1.24 to 5 kg as fed/d/head; 1.81 Mcal/kg DM; 88.32% DM). Thirdly, the total energy requirements remaining were divided by the amount of energy in the feed (1.68 Mcal/kg DM) to obtain the feed intake on a DM basis for each cow. The energy provided by concentrate and PMR was estimated using NASEM Dairy-8 software (v8 R2022.09.02) based on the main dietary ingredients in December.

### Statistical Analysis

Milk yield, milk composition data, and estimated DMI were analyzed as repeated measures with the function “lme” from “nlme” package (Pinheiro, Bates, DebRoy, Sarkar, & R Core Team, 2019), on R software (version 4.0.2.; R Core Team, 2021). The model described by Carpinelli et al. (2021) was modified to the following model:


$$\begin{array}{l} Y_{ijklm}=\mu +A_{o}+D_{i}+P_{j}+DP_{ij}+B_{k}+C_{ijkl}+T_{m}+DT_{im}+DPT_{ijm}+e_{hijklm}, \end{array}$$

where *Y*_*ijklm*_ is the dependent, continuous variable; µ is the overall mean; *A*_*o*_ is the fixed effect of the *o*th of the calving month (*f* = 1,…,12); *D*_*i*_ is the fixed effect of the *i*th treatment (*i* = 1, 2, and 3); *P*_*j*_ is the fixed effect of the *j*th parity (*j* = 2 or higher); DP_*ij*_ is the fixed effect of *i*th treatment by the *j*th parity of the experiment interaction; *B*_*k*_ is the random effect of the *k*th block (*k* = 1,…,39); *C*_*ijkl*_ is the random effect of *l*th cow nested within the *i*th treatment, the *j*th parity, and the *k*th block (*l* = 1,…,*nijk*); *T*_*m*_ is the fixed effect of the *m*th time (period of 15 d) of the experiment (*m* = 1, …,*n*); DT_*im*_ is the fixed effect of the *i*th treatment by the *m*th time of the experiment interaction; DPT_*ijm*_ is the fixed effect of the *i*th treatment by the *j*th parity by the *m*th time of the experiment interaction; and *e*_*ijklm*_ is the residual error. A first-order autoregression covariance structure was used. Pairwise comparisons for the factor Treatment were done using emmeans package (Lenth, 2021), with Tukey adjustment. Statistical significance was declared at ≤0.05 and tendencies at *P* < 0.10. Previous lactation values were added as covariates into the model. A first-order autoregression covariance structure was used.

BHBA levels, BW, BCS, number of services per conception, and calving-to-conception interval were analyzed by the Kruskall–Wallis test using Minitab software (v19.2020.1).

## Results

### Milk Yield and Milk Composition

Daily milk yield was significantly (*P* < 0.01) higher in Y10 than in Y5 and CON (52.0 kg vs. 48.1 and 48.5 kg). ECM was significantly (*P* < 0.05) higher in Y10 than in CON (50.6 kg vs. 47.3 kg) and showed a numerical difference from Y5 (48.2 kg). FCM milk results showed a similar pattern. No significant differences between the treatments were found in milk fat (%), milk protein (%), milk urea, and somatic cell score. The milk yield results are presented in Table 3.

**Table 3:** Effect of yeast probiotic on milk yield in dairy cows (*n* = 39 per treatment) (estimated marginal means and standard errors of the mean, with previous lactation values as covariate)

Item	Y5^1^	Y10^2^	Control	SEM	*P* value
Milk yield, kg/d	48.1^b^	52.0^a^	48.5^b^	1.03	0.008
Fat, %	3.96	3.86	3.79	0.076	NS^3^
Protein, %	3.37	3.32	3.35	0.028	NS
ECM milk, kg/d	48.2^ab^	50.6^a^	47.3^b^	1.04	0.043
FCM milk, kg/d	47.9^ab^	50.7^a^	47.3^b^	1.05	0.033
Urea, mg/100 mL	25.0	24.8	24.6	0.54	NS
Somatic cell score	3.36	3.33	3.49	0.146	NS

^1^Y5 = Control Diet + 5 g/d of yeast probiotic (5 × 10^10^ CFU/cow/d).

^2^Y10 = Control Diet + 10 g/d of yeast probiotic (10 × 10^10^ CFU/cow/d).

^3^NS: statistically non‐significant.

^a,b^Means within a row with different superscripts differ (*P < *0.05).

### BW and BCS

There were no statistically significant differences in BW loss and BCS, although Y5 had numerically lower weight loss during the first 60 and 100 DIM than Y10 and CON (DIM 3 to 90: −13.8, −22.1, −25.3 kg, respectively). The average values are shown in Table 4.

**Table 4:** Effect of yeast probiotic on body weight loss, body condition, ketone bodies in dairy cows (*n =* 20 per treatment) (estimated marginal means and standard errors of the mean)

Item	Y5^1^	Y10^2^	Control	SEM	*P* value
BW loss between DIM 3 to 12 and 55 to 65, kg	−24.6	−29.3	−29.2	4.17	NS^3^
BW loss between DIM 3 to 12 and 90 to 100, kg	−13.8	−22.1	−25.3	7.69	NS
BCS DIM 5	3.2	3.2	3.2	0.07	NS
BCS DIM 60	2.8	2.9	2.7	0.06	NS
BHBA DIM 5, mmol/L	0.83	0.81	1.04	0.143	NS
BHBA DIM 21, mmol/L	0.75	0.76	0.84	0.070	NS

^1^Y5 = Control Diet + 5 g/d of yeast probiotic (5 × 10^10^ CFU/cow/d).

^2^Y10 = Control Diet + 10 g/d of yeast probiotic (10 × 10^10^ CFU/cow/d).

^3^NS: statistically non‐significant.

### Blood Ketone Bodies

Blood ketone levels were generally increased (Duffield et al., 1998) on D5 (Y5: 0.83 mmol/L; Y10: 0.81 mmol/L; CON: 1.04 mmol/L) and on D21 (Y5: 0.75 mmol/L; Y10: 0.76 mmol/L; CON: 0.84 mmol/L), with CON showing the highest values numerically, but no statistically significant differences were found between the groups.

### Reproduction Results

The calving-to-conception interval was numerically shorter in Y5 than in Y10 and CON (99.5 d vs.102.3 and 105.3 d). Also, the number of services per conception was lower in Y5 (2.1 vs. 2.4 and 2.4). Conception success from the first insemination was higher in Y5 and in Y10 groups (39%) than in CON (26%). The results are summarized in Table 5.

**Table 5:** Reproduction results (mean values and standard errors of the mean)

Item	Y5^1^	Y10^2^	Control	SEM	*P* value
Services per conception	2.1	2.4	2.4	0.281	NS^3^
Calving-to-conception interval	99.5	102.3	105.3	7.79	NS
Conceived before 120 DIM	18/24 (75%)	18/26 (69%)	16/22 (73%)		
Conception success from the first insemination	9/23 (39%)	10/26 (38%)	6/23 (26%)		

^1^Y5 = Control Diet + 5 g/d of yeast probiotic (5 × 10^10^ CFU/cow/d).

^2^Y10 = Control Diet + 10 g/d of yeast probiotic (10 × 10^10^ CFU/cow/d).

^3^NS: statistically non‐significant.

### Estimated DMI

No statistically significant differences were found between the groups for total daily DMI estimated using the last equation from NASEM 2021 (22.9, 22.5, and 22.3 kg DM/d, respectively, for Y10, Y5, CON), neither when the approximated amount of concentrate distributed is retrieved from this total, i.e., daily DMI of PMR (19.0, 19.0, and 18.6 kg DM/d). The estimated feed efficiency (ECM divided by estimated DMI) was numerically the highest for the Y10 group (2.22, 2.15, and 2.12, respectively for Y10, Y5, and CON). The results are presented in Table 6.

**Table 6:** Effect of yeast probiotic on estimated daily dry matter intake based on 263 observations (kg DM/d, estimated marginal mean and standard errors of the mean)

Item	Y5^1^	Y10^2^	Control	SEM	*P*
Estimated total DMI, kg DM/d (NASEM 2021 model)	22.5	22.9	22.3	0.49	NS^3^
Estimated DMI of PMR, kg DM/d (total—concentrate; NASEM 2021 equation)	19.0	19.0	18.6	0.42	NS

^1^Y5 = Control Diet + 5 g/d of yeast probiotic (5 × 10^10^ CFU/cow/d).

^2^Y10 = Control Diet + 10 g/d of yeast probiotic (10 × 10^10^ CFU/cow/d).

^3^NS: statistically non‐significant.

## Discussion

This study investigated the dietary supplementation of two different doses of yeast probiotic (*S. cerevisiae*, strain CNCM I-4407, 10^10^ CFU/g; Actisaf Sc 47; Phileo by Lesaffre, Marcq-en-Baroeul, France), as compared with an unsupplemented control, in high-producing dairy cows in early and peak lactation. The highest ECM daily yield was achieved with the high dose group (10 × 10^10^ CFU/cow/d, Y10) which also showed a higher estimated feed efficiency. The low-dose group (5 × 10^10^ CFU/cow/d, Y5) had the lowest average loss of BW during the first 100 d of lactation, however, the difference was not statistically significant. Reproduction results (calving-to-conception interval, number of services per conception, conception success from the first insemination) were generally the best in the yeast probiotic group.

Many studies have reported an increase in milk yield as a result of yeast probiotic supplementation (Desnoyers et al., 2009; Ondarza et al., 2010; Salvati et al., 2015; Kumprechtová et al., 2019; Perdomo et al., 2020). Milk yield increase in response to yeast probiotic supplementation may be attributed to increased feed efficiency via improved nutrient digestibility (Marden et al., 2008; Ferraretto et al., 2012; Jiang et al., 2017a; Julien et al., 2018; Perdomo et al., 2020) resulting from stabilized microbial community in the rumen (Bach et al., 2007; Ondarza et al., 2010; Jiang et al., 2017b). Proposed modes of action of supplemental yeast *S. cerevisiae* on ruminal microbiota composition include modification of the ruminal environment through oxygen scavenging which leads to a decrease in redox potential favoring the growth of anaerobic cellulolytic bacteria in the rumen (Newbold et al., 1996; Marden et al., 2008; Jiang et al., 2017b). The cellulolytic flora is also supported by an increase in pH and mitigation of postprandial pH drops in cows receiving a high-starch diet (Marden et al., 2008), which is linked with increased activity of lactate utilizing bacteria (Chaucheyras et al., 1996; Rossi et al., 2004; Pinloche et al., 2013; Huang et al., 2017). Changes in ruminal microbiota profile lead to increased total volatile fatty acid (**VFA**) concentration (Hasunuma et al., 2016; Kumprechtová et al., 2019; Oh et al., 2019), and decreased ratio of acetic to propionic (Marden et al., 2008; Kumprechtová et al., 2019; Meller et al., 2019) and increased efficiency of energy usage (Dias et al., 2018).

In the present study, the dose effect of yeast probiotics on ECM yield was clearly visible (CON: 47.3, Y5: 48.5, Y10: 50.9 kgs/d). The dose effect on milk yield is variable among other studies, which may be due to differences in acidogenicity of diet (Bach et al., 2007; Marden et al., 2008), stage of lactation (Poppy et al., 2012), level of production, DIM of cows (Erasmus et al., 2005), yeast probiotic viability, dosage, etc. A similar dose effect of yeast probiotics on milk yield was seen in our previous study (Kumprechtová et al., 2012) comparing 0, 1 × 10^10^, and 5 × 10^10^ CFU/cow/d of yeast probiotics (5 × 10^9^ CFU/g, *S. cerevisiae*, strain CNCM I-4407) supplemented to high-producing dairy cows from 21 to 112 d postpartum, resulting in average daily milk yield of 41.9, 43.5, and 43.9 kg, respectively. On the other hand, Ferraretto et al. (2012) detected no effects of live yeast dose (0, 2 × 10^10^, and 4 × 10^10^ CFU/cow/d, *S. cerevisiae*, strain NCYC 996, Procreatin-7, Phileo by Lesaffre) on milk yield in dairy cows; however, DM, OM and NDF digestibility was greater for 4 × 10^10^ CFU/cow/d than for 2 × 10^10^, and 0. Acharya and Dhital (2018) observed a significant improvement in milk yield only with 3 × 10^10^ CFU (2 g) live yeast *S. cerevisiae* (1.5 × 10^10^ CFU/g) per kg feed but not with 7.5 × 10^9^ CFU (0.5 g) and 1.5 × 10^10^ CFU (1 g) over the unsupplemented control in low-producing Nepal dairy cattle. Mašek et al. (2008) observed a positive dose effect of 3 × 10^9^ CFU g and 6 × 10^9^ CFU g of yeast culture (*S. cerevisiae,* NCYC 1026—Yea-Sacc 1026) on FCM yield in dairy ewes. On the other hand, Al Ibrahim et al. (2010) who used 2.5 × 10^9^ CFU and 10 × 10^9^ CFU of *S. cerevisiae* NCYC 1026 did not observe any effects on milk and milk composition.

In this study, BW loss till 100 DIM was numerically lower in the Y5 group than in Y10 and Control (−13.8, −22.1, and −25.3 kg, respectively). Julien et al. (2017) observed significantly higher BCS 8 d postpartum for cows supplemented with yeast probiotics during the transition period (2.94 vs. 2.42). Muruz and Gül (2020) and Tristant and Moran (2015) did not find any such effects. However, Zhu et al. (2016) reported that the mean BCS of dairy cows fed 120 g/d of live yeast was higher than for both the control and 240 g/d yeast probiotic groups due to improved net energy balance during heat stress. It might be associated with a higher milk production when supplemented with a higher dose of yeast probiotics.

In the present study, yeast probiotic administration was associated with better fertility results. Better feed efficiency and hence a lower energy deficit when beginning lactation may improve reproductive function in dairy cows (Julien et al., 2018). Julien et al. (2018) in their study comprising 14 dairy cattle farms administered yeast probiotic (*S. cerevisiae* CNCM I-4407 1 × 10^10^ CFU/g) at 5 × 10^10^ CFU/cow/d over the 6 wk around calving time evaluated reproduction parameters. The use of daily supplementation with probiotic yeast resulted in a significant improvement of 4 points on average in the success rate of artificial insemination and of 5 points in the success rate of first artificial insemination in multiparous dairy cows. In their previous study, Julien et al. (2017) have demonstrated that the success rate at the first AI is generally better in dairy cows receiving a high dose (10 × 10^10^ to 20 × 10^10^ CFU/cow/d) of yeast probiotics. Nasiri et al. (2018) reported greater plasma IGF-I, E-17β, and P4 concentrations, larger ovulatory follicles, shorter estrous cycles, and improved reproductive performance in high-producing dairy cows given 6 × 10^10^ CFU (4 g) of live yeast/cow/d, exposed to heat stress.

In the present study, the lower dose of yeast probiotic (Y5) produced better fertility results than the higher dose (and Control). The calving-to-conception interval was numerically shorter in Y5 than in Y10 and CON (99.5 d vs. 102.3 and 105.3 d). Also, the number of services per conception was lower in Y5 (2.1 vs. 2.4 and 2.4). The success rate of the first artificial insemination was the highest in the yeast probiotic groups (39%, 38%) than in the control group (26%). Reproduction performance is closely related to energy balance. In high-yielding cows the energy output by production can be at the expense of conception capability (Courtheix, 2016). Our assumption is that lower energy output by milk and smaller weight loss due to yeast probiotic supplementation in Y5 may have contributed to better fertility. Furthermore, the improvement in milk yield, feed efficiency and fertility in other studies with supplementation of *S. cerevisiae* CNCM I-4407 at 10 × 10^10^ CFU/cow/d have shown to reduce carbon emission by 5.5% in a full scope life-cycle assessment (LCA conducted under ISO14040/14044 in revision, Blonk Consulting, 2023).

## Conclusions

This field study confirms the beneficial effects of optimizing the yeast probiotics dose for high-producing dairy in early and peak lactation to improve their productive and reproductive performance. Accurate dosing of yeast probiotics according to dairy cow’s individual needs may support precision feeding strategies to optimize feed efficiency. The recommended dose (5 × 10^10^ CFU/cow/d) of yeast probiotic, compared to the control group, showed a numerical ECM production increase, better retention of BW in early lactation, and better reproduction results indicating a better use of energy from the diet. In this case, with high-producing early lactation animals, we suggested that 10 g of yeast probiotic (10 × 10^10^ CFU/cow/d) would be more suitable. With the higher dosage, we observed higher ECM milk production without reducing the conception ability and suggested a higher efficiency of DMI conversion to ECM. Future studies will have to reinforce that the dosage modulation of *S. cerevisiae*, strain CNCM I-4407, according to the specific needs of the dairy cows, may be seen as a solution for a more sustainable dairy production focusing on feed and resource efficiency.
